# Computational Fluid Dynamic Analysis of Fluid Motion and Volumetric Gas–Liquid Mass Transfer in Agitated Platelet Concentrate Storage

**DOI:** 10.1002/biot.70177

**Published:** 2026-01-08

**Authors:** Dean Pym, Amanda J. Davies, Jessica O. Williams, Christine Saunders, Chloë E. George, Allan Mason‐Jones, Philip E. James

**Affiliations:** ^1^ Cardiff Metropolitan University, Centre of Cardiovascular Health and Ageing, Llandaff Campus Cardiff UK; ^2^ Component Development and Research Laboratory Welsh Blood Service, Ynysmaerdy Pontyclun UK; ^3^ Cardiff School of Engineering, Cardiff University, Queen's Buildings Cardiff UK

## Abstract

Computational fluid dynamics (CFD) offers a powerful tool in characterizing the complex biophysical environment inducing by dynamic storage conditions, providing insights often beyond the reach of conventional experimental approaches. As our understanding of platelet (PLT) biology has advanced, increased attention has been directed toward mechanical stresses, attributing shear forces encountered during collection, processing, and storage to an acceleration decline in PLT concentrate (PC) quality. CFD simulations using the volume of fluid model were used to simulate PC storage under varying agitation frequencies. Key parameters assessed include fluid velocity, wall shear stress (WSS), and gas–liquid mass transfer. Agitation increased fluid velocity and WSS while preserving the temporal symmetry characteristic of sinusoidal motion. Enhanced oxygen transfer was observed in open‐top containers; however, when accounting for the gas permeability of storage materials, oxygen availability was ultimately constrained by container permeability rather than fluid motion. These results highlight the dual role of agitation: promoting oxygen transfer while simultaneously introducing mechanical stress that may contribute to PLT storage lesions. Importantly, since oxygen supply is limited by container permeability, reducing agitation could minimize shear‐induced PLT damage without compromising oxygenation. Future optimization strategies may involve modifying storage container geometry or permeability to further improve oxygen delivery during storage.

## Graphical Abstract and Lay Summary

Platelets (PLTs) are small blood cells that play a vital role in stopping bleeding. After donation, they are stored in special bags, but their quality decreases over time. One reason for this decline is the mechanical stress they experience during collection, processing, and storage. PLT bags are often agitated (gently shaken) to help maintain oxygen supply, but this movement has also been suggested to increase stress on the cells. Using computer simulations, we studied how agitation affects fluid flow, mechanical stress, and oxygen transfer in stored PLTs. We found that agitation increases fluid movement and stress on PLTs. It also improves oxygen transfer when PLTs are stored in open containers. However, in standard storage bags, oxygen availability is mostly determined by the material of the bag, not by agitation. These results suggest that reducing agitation could help to protect PLTs from stress without reducing oxygen supply. Future improvements in PLT storage may come from designing bags with better materials or shapes to optimize oxygen transfer, rather than relying heavily on agitation.

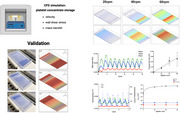

## 1 Introduction

Platelet concentrates (PCs) are an essential component of transfusion medicine, commonly administered to patients to manage the bleeding risk associated with bleeding disorders, traumatic injury, and during major surgeries. To ensure the therapeutic efficacy of PCs is maintained, PCs are typically stored under controlled conditions for up to 7 days, requiring storage at room temperature, in a gas permeable storage container, under constant agitation. Despite this, platelets (PLTs) are sensitive to their biophysical environment, and storage‐induced stresses can lead to a range of deleterious changes collectively known as the PLT storage lesion (PSL) [1]. These changes are onset by PLT activation and result in shape change, cytoskeletal reorganization, and shedding of key adhesion receptors, with receptors for thrombin (PAR1, PAR4), collagen (GPVI), and von Willebrand factor (CD42b) being shed during PC storage [2, 3, 4, 5, 6, 7]. Overall, these changes result in reduced in vitro function, increase clearance in the spleen and liver posttransfusion, and ultimately reduce the prophylactic success of the transfused product [8, 9, 10].

Agitation was originally introduced to PC storage protocols to improve oxygen (O_2_) permeability by maintaining an O_2_ gradient across the bag film [11]. Early studies showed nonagitated PCs had reduced posttransfusion recovery (23%) and shorter circulation half‐life (2.6 days), whereas PCs stored with agitation had significantly higher posttransfusion outcomes (36% recovery; 3.6 days half‐life) [12]. These improvements were attributed to enhanced gas exchange across the storage bag. As our understanding of PLT biology has advanced, increased attention has been directed toward mechanical stresses, attributing shear forces encountered during collection, processing, and storage to an acceleration decline in PC quality [13, 14, 15]. Shear is defined by both the shear rate, change in velocity between two adjacent fluid microlayers divided by their distance, and shear stress, the magnitude of tangential force applied coplanar to the flow region [16, 17]. In liquids, shear stress can be described by −μ(*dv*/*dh*), where *μ* is the dynamic fluid viscosity, *ν* is the fluid speed, and *h* is the height above the face of a material [18]. Since shear stress is governed by the rate and duration of flow, agitation is the sole driver of shear exposure during PC storage. Despite this, the physical environment PCs experience during storage as a consequence of agitation remains poorly characterized.

Computational fluid dynamics (CFD) is a numerical method used to predict fluid flow by solving the equations that govern fluid motion [19], enabling detailed visualization and quantification of velocity fields, wall shear stress (WSS) distribution, and volumetric gas–liquid mass transfer rates. Because of its ability to noninvasively predict internal fluid dynamics, CFD is routinely used in the design and optimization of bioreactors, with a growing number of studies applying computational methods to small scale biological systems [20, 21, 22]. Several studies have investigated fluid dynamics in systems relevant to biological processing and culture using CFD. Zhang et al. examined velocity distributions and O_2_ mass transfer in shake flasks, demonstrating how agitation affects gas exchange in fermentation systems and cell growth in mammalian cell culture [23]. Mehmood et al. applied CFD under laminar flow assumptions to study the relationship between power dissipation and gas–liquid volumetric mass transfer (*K*_L_ *A*), with culture performance in systems cultivating filamentous microorganisms [24]. Liu et al. focused on determining the shear sensitivity threshold of plant cells cultured in shaken flask bioreactors [25]. More recently, Svay et al. investigated fluid behavior in wave bag bioreactors, quantifying *K*_L_ *A*, mixing times, and velocity distribution across varying rocking speeds and angles [26].

Collectively, these studies highlight the value of CFD in characterizing mechanical stress and mass transfer in biological systems. Although the application of CFD to inform PC storage practices remains unexplored, shake flask and wave‐bag bioreactors exhibit flow regimes analogous to those in PC storage, including slow, laminar circulation, recirculation zones, oscillatory motion, and periodic shear, all of which influence mixing and mass‐transfer behavior. Likewise, previous studies have begun to explore PLT behavior in flow contexts using related approaches. Torres et al. examined fluid motion and shear forces in agitated PC storage bags using experimental bead tracking, providing early insight into how agitation influences local flow environments [27]. Han et al. reviewed numerical models for shear‐induced PLT activation, establishing the importance of flow‐induced mechanical cues on PLT physiology [28]. Conceptually, these approaches are analogous to CFD studies in shake flasks and wave‐bag bioreactors, where velocity distributions, *K*_L_ *A*, and shear forces have been quantified to optimize culture performance. However, prior work has not captured the coupled fluid‐mechanical and diffusive transport phenomena that govern gas exchange and shear exposure during PC storage, with reference to agitation and container gas permeability. This study aimed to apply CFD to characterize the biophysical environment within the PC storage container under varying agitation frequencies. By capturing temporal changes in velocity, WSS, and volumetric gas–liquid mass transfer, this work offers a foundational understanding of the agitation‐induced mechanical environment, supporting the optimization of PC storage protocols to minimize the shear‐induced PSL in PCs reserved for neonatal transfusion [15].

## 2 Methods

### 2.1 Computational Fluid Dynamics

CFD was used to calculate the velocity field and gas–liquid free surface of two clearly separated and noninterpenetrating phases. With this, the volume of fluid (VOF) method was suitable to predict the free surface flow and container hydrodynamics due to its effectiveness in tracking the interface between two immiscible phases in transient multiphase flow simulations.

#### 2.1.1 The Volume of Fluid (VOF) Approach

The VOF method employs a phase fraction function, “*r* (*x*, *t*)”, which varies between two distinct phases, 0 and 1, with intermediate values representing the interface region. The VOF method has been described comprehensively elsewhere [23, 29, 30, 31]. The governing equations are concisely described below (Equation 1):

(1) $$\frac{\partial r\left(x,t\right)}{\partial t}+\nabla \cdot \left[ur\left(x,t\right)\right]=0$$

where “*t*” denotes time, and “*u*” is the velocity vector. This equation governs the advection of the phase fraction and determines the movement of the interface. When solving Equation (1), the velocities of the two phases are assumed to equilibrate over short distances, and the conservation of mass (Equation 2) and momentum (Equation 3) equations are thought to be homogeneous; therefore, assuming transient flow:

(2) $$\frac{\partial p}{\partial t}+\nabla \cdot \left(pu\right)=0$$

(3) $$\frac{\partial \left(pu\right)}{\partial t}+\nabla \left(puu\right)=-\nabla p-\nabla \cdot \tau +F_{s}$$

where“*p*” denotes pressure, “*τ*” is the viscous stress tensor, and “*F_s_*” represents surface tension, which is represented as a body force. The material properties, such as density *ρ* (Equation 4) and viscosity *μ* (Equation 5), are computed as weighted averages based on the phase fractions. Where *r*1 and *r*2 are the phase fractions of Fluids 1 and 2, respectively, and *ρ*1, *ρ*2, *μ*1, and *μ*2 are their corresponding densities and viscosities.

(4) p=r1p1+r2p2

(5) μ=r1μ1+r2μ2

Surface tension effects are incorporated as a body force in the momentum equation, typically modeled using the Continuum Surface Force (CSF) approach [32]. This method represents surface tension as a volumetric force concentrated at the interface between phases (Equation 6), where *σ* is the surface tension coefficient, and *κ*(*x*) is the interface curvature, given by Equation (7). The vector normal “*n*” is defined in Equation (8).

(6) $$F_{s}=\sigma k\left(x\right)r\left(x,t\right)$$

(7) $$k=\frac{1}{n}\left[\left(\frac{n}{\left|n\right|}\cdot \nabla \right)\left|n\right|-\nabla \cdot n\right]$$

(8) $$n=\nabla r\left(x,t\right)$$

#### 2.1.2 Computational Domain and Boundary Conditions

Numerical simulations of the fluid flow equations (Equations (1), (2), (3), (4), (5), (6), (7), (8)) were achieved using ANSYS fluent computational fluid dynamic software (ANSYS fluent). A sweep mesh, divided into 452.4k elements and 477.2k nodes, was used in the analysis. A no‐slip wall boundary condition was applied to each solid wall, and the pressure outlet was set at the top surface of the geometry. The multiphase solver used the PISO algorithm for coupling pressure and velocity, under the assumption of laminar flow supported by Reynolds number estimates (Re < 2300). Likewise, several validated VOF‐laminar studies in shake flasks and wave‐bag bioreactors have been shown to accurately capture the expected fluid motion [23, 24, 26]. PISO was designed specifically for transient, unsteady flows; it performs multiple correction steps (pressure corrections and velocity adjustments) within a single timestep, which improves temporal accuracy and reduces the number of iterations per step. This makes PISO faster and more stable for free‐surface multiphase flows, where pressure–velocity coupling changes rapidly. In the analysis of oscillatory linear motion, representative of agitation, water (density: 997 kg/m^3^, viscosity: 1 cP), and air (density: 1.2 kg/m^3^, viscosity: 0.018 cP) were applied for Phases 0 and 1, respectively. The acceleration of the container due to oscillatory linear motion was described as Equation (9); where *w* is the angular frequency.

(9) $$\mathrm{Acc}\left(x\right)=-a*w^{2}*\mathrm{Sin}\left(w*t\right)$$

**FIGURE 1:**
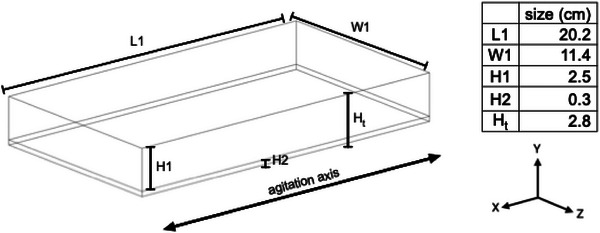
Geometry overview. Shows the geometry presented using a wire frame, with L1 describing the length of the container, W1 describing the width of the container, H1 representing the height of the container filled with air, and H2 representing the height of the liquid layer. The pressure outlet was set to the top surface of the geometry, and a no‐slip wall boundary condition was applied to each other the solid wall. The oscillatory motion was implemented by applying an acceleration described in Equation (9) in the *X* axis, while gravitational acceleration acted in the vertical *Y* axis. Mesh quality metrics and a mesh independence study are provided in the Supporting Information.

For experimental comparisons, a container matching the dimensions specified in Figure 1 was filled with approximately 65 mL of distilled water containing 0.01% trypan blue dye (BDH Chemicals Ltd, C.I. 23850). The container was positioned on a flatbed agitator under standard blood banking conditions (22 ± 2°C) with a constant agitation horizontal displacement of ±2 cm and agitated at frequencies ranging from 20 to 60 rpm.

#### 2.1.3 Model Validation

To ensure the reliability of the numerical simulations, the model was verified through mesh independence and experimental validation. Mesh independence was assessed using four progressively refined meshes were compared (Figures S1–S5). Mesh II (452.4k elements) provided the best resolution of the liquid–gas interface, which was critical for accurately capturing interfacial area dynamics (Figure S2). Negligible differences in bulk flow parameters were shown between Mesh III (65.52k elements) and Mesh IIII (Figures S3–S5), confirming numerical convergence. Mesh quality was consistently high across all cases (minimum orthogonal quality = 1.0; maximum aspect ratio = 2.3–4.3). Therefore, Mesh IIII was chosen for the final simulations due to its resolution of the liquid–gas interface.

For experimental validation, fluid motion was measured using a particle tracking method as described elsewhere [15], with minor modifications. Videos were contrast enhanced and analyzed in ImageJ using the Trackmate plugin (LoG detector, simple LAP tracker). Bead velocities were calculated between consecutive frames to obtain instantaneous fluid speeds over time. A minimum of 20 beads were analyzed over ∼600 frames. Pixel‐to‐distance calibration was based on the known bag dimensions, and velocities were converted to cm/s.

#### 2.1.4 Mass Transfer

The volumetric liquid‐phase mass‐transfer coefficient (*K*_L_ *A*) was used to account for the combined effects of diffusion and convection at the gas–liquid interface, following Higbie's penetration theory (Equation 10). Where *K*_L_ represents the rate of molecular diffusion through the gas‐liquid interface, “*D*” represents the diffusion coefficient, given as the diffusion coefficient of water (*D*_water_ = 2.42 × 10^−9^ m/s), and “*t*_C_” represents the contact time, calculated as the width of the storage container along the agitation direction (*W*1), by the averaged local velocity along the interface (*V*_interface_), as described elsewhere [26, 33]. “*A*” represents the area of the gas–liquid interface, obtained from the CFD simulation calculated by the summation of the area of the isosurface with a phase fraction of 0.5.

(10) $$K_{L}=2{\left(\frac{D}{\pi t_{C}}\right)}^{\frac{1}{2}}\;\mathrm{with}\;t_{C}=\frac{W1}{V_{\mathrm{interface}}}$$

To consider the influence of the storage container on O_2_ mass transfer, the total mass transfer coefficient (*K*_Total_) can be expressed by a resistance in series model (Equation 11), where the total resistance (*R*_total_) is defined as the reciprocal of *K*_total_, comprising the summative resistance of the storage container (*R*_bag_) and the liquid (*R*_L_). *R*_bag_ and *R*_L_ can be defined by the reciprocal of storage container mass transfer coefficient (*K*_bag_) and the liquid mass transfer coefficient (*K*_L_), respectively. *K*_bag_ was calculated from the O_2_ transmission rate of two different bag materials, TOTM‐PVC and polyolefin, provided as ∼690 and ∼2000 cm^3^/m^2^/24 h, respectively.

(11) $$R_{\mathrm{total}}=\frac{1}{K_{\mathrm{total}}}=\frac{1}{K_{\mathrm{bag}}}+\frac{1}{K_{L}}=R_{\mathrm{bag}}+R_{L}$$

## 3 Results

### 3.1 Validation of the Computational Model With Experimental Fluid Motion

**FIGURE 2:**
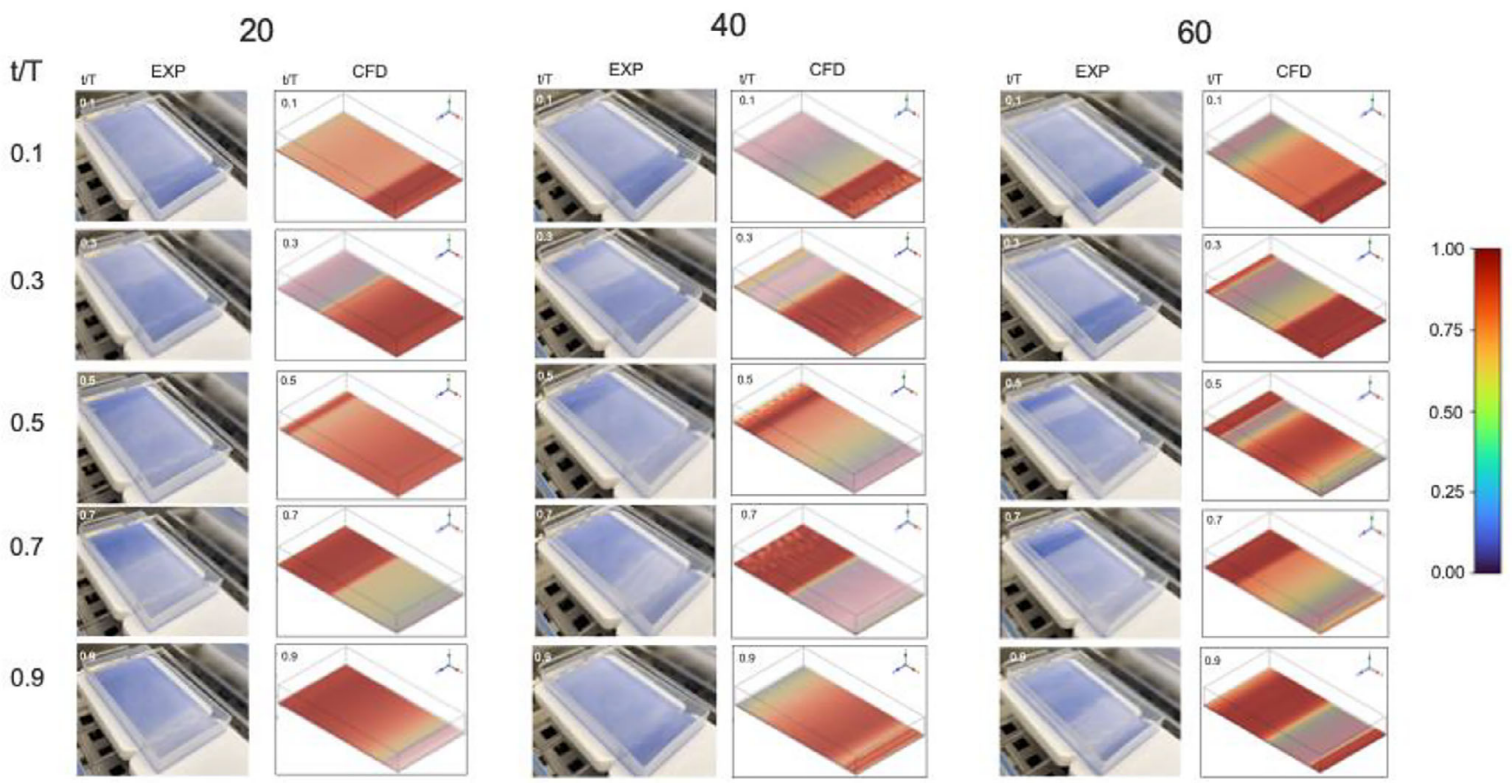
Comparison of experimental and computational fluid motion during agitated PC storage. Visualization was done at 20, 40, and 60 rpm at normalized time points (*t*/*T* = 0.1, 0.3, 0.5, 0.7, and 0.9) across the oscillation cycle. Rows represent different time points within the cycle, and columns represent different agitation frequencies. EXP represents experimental bulk fluid motion, while CFD represents the simulated water volume fraction distribution. The color scale represents the volume fraction, with blue representing air (=0), and red indicating water (=1). CFD, computational fluid dynamics; PC, platelet concentrate.

**FIGURE 3:**
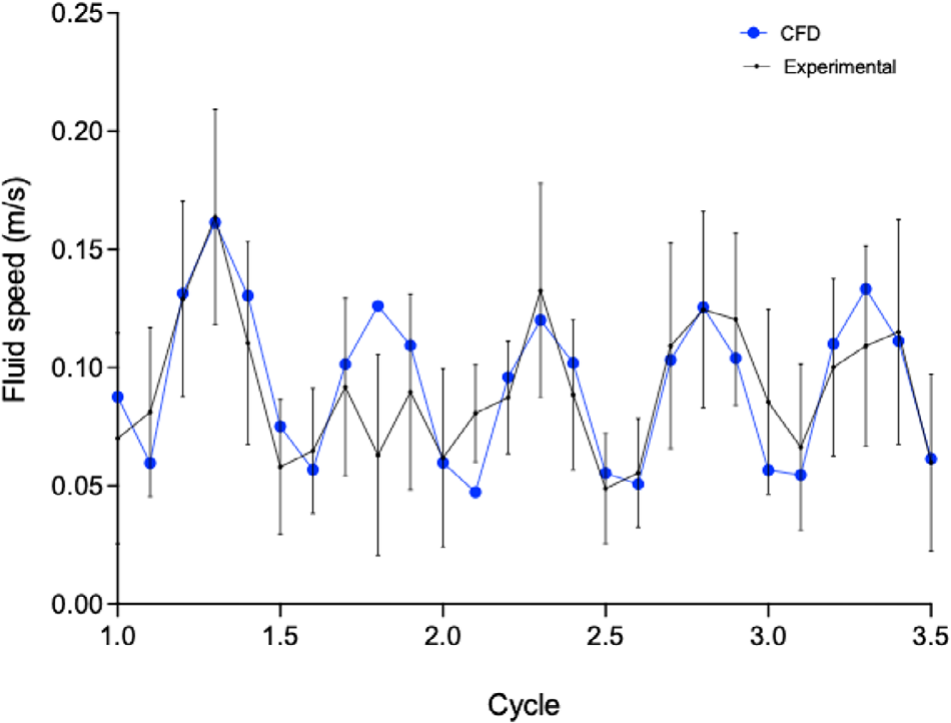
Validation of computational fluid dynamics (CFD) predictions with experimental bead tracking analysis. Experimental bead‐tracking measurements (black line, mean ± 95% CI) are compared with CFD‐predicted volume‐averaged fluid velocities (blue line) under laminar flow conditions.

To assess the accuracy of the CFD simulation, a comparison was done between the fluid motion of the simulated water phase fraction in the fluid domain and experimental bulk fluid motion, at agitation frequencies of 20, 40, and 60 rpm (Figure 2). All agitation frequencies demonstrate temporal symmetry reflective of the sinusoidal nature of the agitation mode. At 20 rpm, fluid motion appears more gradual with limited wave formation and minimal deformation. In contrast, at 40 and 60 rpm, the fluid responses become more dynamic, resulting in sharper interface gradients, sharper leading edges and deeper troughs as the fluid is displaced. Counter‐propagating wavefronts develop at both 40 and 60 rpm; however, at 60 rpm, the counter‐propagating waves appear to interact, leading to transient interface flattening and distortion. The CFD model captures this phenomenon effectively, revealing a momentary decrease in interface steepness and reorganization of the fluid layer. At all agitation frequencies, the CFD predictions qualitatively matched the experimentally observed bulk fluid motion and more nuanced intra‐cycle behaviors that emerge at higher agitation frequencies over the oscillation cycle. Quantitative comparison using bead tracking analysis further supported the model's validity, with a strong Pearson correlation between simulated and experimental velocity fields (*r * =  0.818, *p* << 0.0001; Figure 3), further supporting the model's reliability for analyzing fluid behavior across varying agitation regimes.

### 3.2 Fluid Behavior During Agitated PC Storage

**FIGURE 4:**
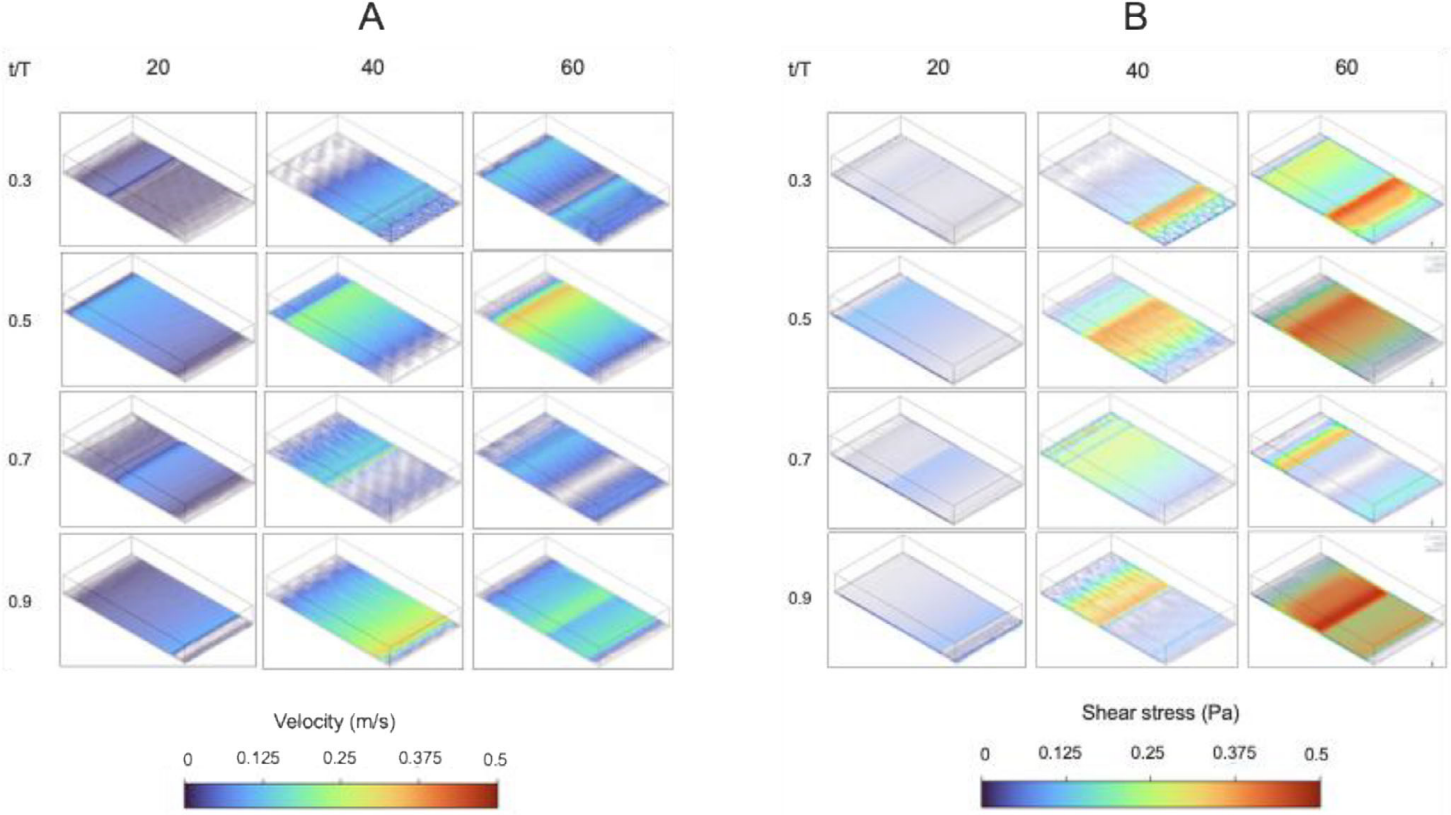
Fluid behavior distributions during agitated platelet concentrate (PC) storage. (A) Represents the spatial distribution of velocity and (B) represents the spatial distribution of shear stress (SS) during agitated PC storage. Visualization was done at 20, 40, and 60 rpm at normalized time points (*t*/*T* = 0.3, 0.5, 0.7, and 0.9) across the oscillation cycle. Each row represents a different time point within the cycle, while each column represents a different agitation frequency. Magnitudes are shown on a color scale, with blue representing low velocity or SS, and red indicating high velocity or SS.

Following validation of the CFD model against experimental observations, the simulation was used to examine the internal fluid behavior under different agitation frequencies. Specifically, velocity, WSS, and pressure distributions were assessed at agitation frequencies of 20–60 rpm. Figure 4A presents the spatial velocity distribution at normalized time points (*t*/*T* = 0.3, 0.5, 0.7, and 0.9) across three agitation frequencies. At 20 rpm, the velocity field appears relatively uniform, with velocity peaking at ∼0.12 m/s during steady transients. At 40–60 rpm, the flow exhibits pronounced velocity gradients and localized fluctuations, particularly during the second half of the oscillation (*t*/*T* = 0.5–0.9). The peak velocity during 40 rpm agitation is ∼0.32 m/s, while the peak velocity during 60 rpm agitation is ∼0.45 m/s. At all agitation frequencies, the maximum velocity is present just behind the wave peak. Over the oscillation cycle, the velocity reaches a maximum at the maximum displacement of the oscillation (*t*/*T* = 0.5 and 0.9).

WSS distributions for the same conditions are presented in Figure 4B. As expected, higher agitation rates result in increased WSS, particularly at timepoints where flow reversal induces steep velocity, and around local regions characterized by high velocity. At 60 rpm, peak WSS values reach up to ∼0.5 Pa, showing the greatest distribution of high WSS at *t*/*T* = 0.5 and 0.9. This correlates with the maximum displacement of the oscillation. Forty revolutions per minute displays a similar WSS spatial distribution, with the greatest distribution of high WSS at *t*/*T* = 0.5 and 0.9. However, the WSS magnitude is reduced at 40 rpm, with WSS peaking at ∼0.38 Pa, and showing a more uniform distribution of WSS at *t*/*T* = 0.5 and 0.7, compared to 60 rpm. In contrast, at 20 rpm, WSS appeared relatively low and uniform throughout the cycle, peaking at ∼0.12 Pa.

**FIGURE 5:**
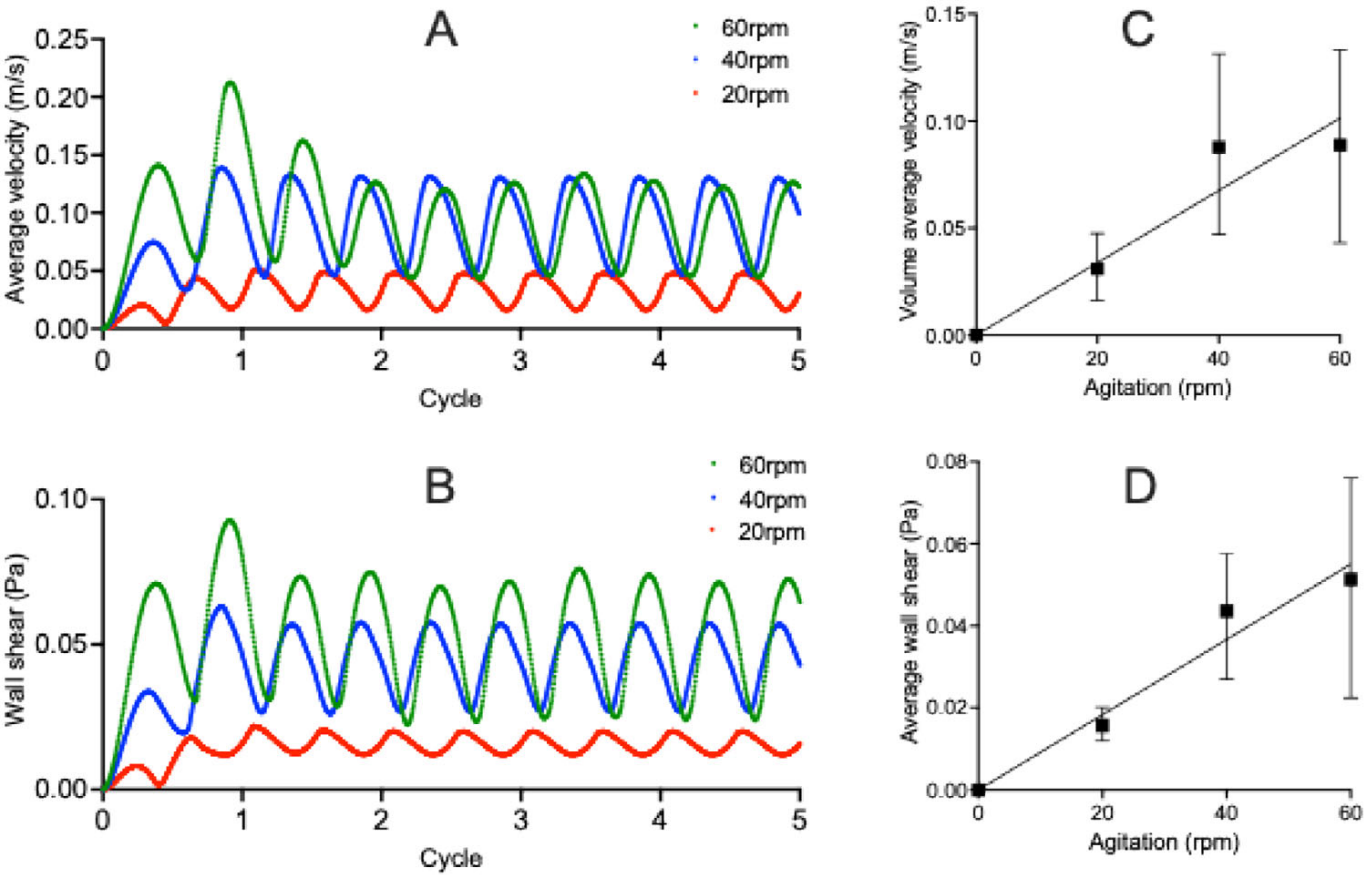
Temporal variations in velocity and WSS during agitated PC storage. Temporal profiles for volume‐averaged fluid velocity (A) and volume‐averaged WSS (B) were monitored over five oscillation cycles at 20 rpm (red), 40 rpm (blue), and 60 rpm (green) agitation. Plots against agitation frequency for velocity (C) and WSS (D) were averaged across steady transient oscillation cycles (cycles 2–5) with error bars denoting variance. Statistical significance was inferred by linear regression. PC, platelet concentrate; WSS, wall shear stress.

To quantify the time‐dependent behavior of these mechanical behaviors, volume‐averaged fluid velocity and WSS were monitored across five complete oscillation cycles (Figure 5A,B). Both velocity and WSS demonstrate clear periodicity, synchronized with the imposed sinusoidal motion. Notably, peak values occur during the initial cycle, followed by stabilization. This is likely due to the dissipation of start‐up transients and the establishment of a steady transient state. The relationship between agitation frequency and mean mechanical metrics was further investigated using linear regression. Strong positive correlations were identified for both averaged velocity (Figure 5C) and WSS (Figure 5D). For velocity, the linear regression analysis showed the slope to be significant (*F* (DFn, DFd) = 18.85 (1, 2), *p* = 0.0492) with a best fit line described by: *Y* = 0.001617*X (*R* ^2^ = 0.9041). Linear regression analysis also showed the slope to be significant for WSS (*F* (DFn, DFd) = 48.45 (1, 2), *p* = 0.02) with a best fit line described by: *Y* = 0.0009084*X (*R* ^2^ = 0.9604). Residual analysis confirmed the absence of systematic bias across agitation speeds (20–60 rpm) (Figure S6).

### 3.3 Gas–Liquid Mass Transfer

**FIGURE 6:**
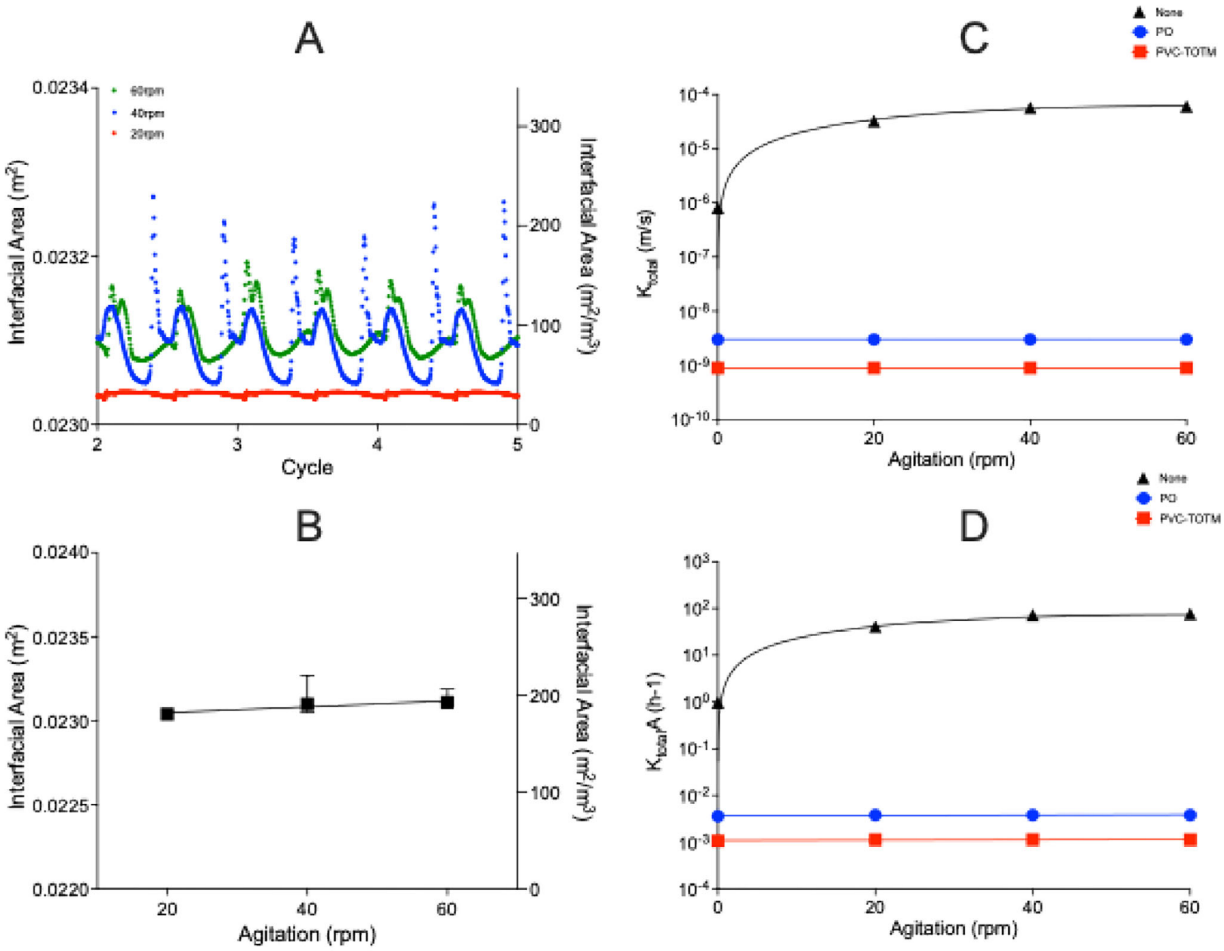
Gas–liquid mass transfer during agitated platelet concentrate (PC) storage. (A) interfacial area was monitored over three oscillation cycles (cycles 2–5) at 20 rpm (red), 40 rpm (blue), and 60 rpm (green) agitation. Plots against agitation frequency for (B) interfacial area were averaged across the three oscillation cycles with error bars denoting variance. Statistical significance was inferred by linear regression. Plots against agitation frequency for *K*_total_ (C), and *K*_total_ *A* (D) were averaged across the three oscillation cycles. Statistical significance was inferred either by linear or nonlinear regression. Container denotations were as follows: “None” = open top container; “PO” = polyolefin container; “PVC‐TOTM” = PVC‐TOTM container.

The variation in interfacial area over the oscillation cycles at 20–60 rpm agitation is shown in Figure 6A. As initial transient effects were evident during the first two cycles for velocity and WSS, which seemed to stabilize on the onset of Cycle 3, quantitative comparisons were made once oscillatory dynamics reached steady transients. Clear differences in the magnitude and temporal pattern of interfacial area were observed across agitation frequencies. Forty revolutions per minute exhibited the greatest variation in interfacial area over time, with pronounced peaks and troughs reflective of cyclic oscillation. This was followed by 60 rpm, which showed moderately dampened fluctuations. On the other hand, 20 rpm showed a relatively flat profile, representing minimal variation in interfacial area over time. To exclude artifacts from mesh resolution at the liquid–air interface, the mesh independence study (Figures S1–S2) confirmed that Mesh IIII resolution of the liquid–gas interface (452k elements, 477k nodes) yielded the sharpest interface and minimal numerical diffusion. The relationship between agitation frequency and mean interfacial area was further investigated using linear regression (Figure 6B). The linear regression analysis showed the slope to be nonsignificant (*F* (DFn, DFd) = 5.88 (1, 1), *p* = 0.2490).

In contrast, *K*_total_ demonstrated a nonlinear relationship with agitation frequency when stored in an open‐top container (where 1/*K*_total_ = 1/*K*_L_), fitting a second order polynomial curve (*Y* = *B*0 + *B*1 * *X* + *B*2 * *X* ^2^). The fitting values for *K*_L_ were as follows: *B*0 = 6.107 × 10^−8^, *B*1 = 2.064 × 10^−6^, and *B*2 = −1.749 × 10^−8^ (*R* ^2^ = 0.9951; Figure 6C). Alternatively, when considering the gas permeability of a storage container (where 1/*K*_total_ = 1/*K*_bag_ + 1/*K*_L_), composed of either polyolefin or PVC plasticized with TOTM, *K*_total_ showed no apparent relationship to agitation frequency, with linear regression analysis showing no significant slope for polyolefin (*F* (DFn, DFd) = 3.12 (1, 2), *p* = 0.2194) and PVC‐TOTM (*F* (DFn, DFd) = 3.115 (1, 2), *p* = 0.2196) as agitation frequency increased. However, a significant difference was shown in the intercepts between the two materials (*F* (DFn, DFd) = 760283 (1, 5), *p* < 0.0001).

A similar pattern was shown with *K*_total_ *A*. In an open top container (where *K*_total_ *A* = *K*_L_ *A*), *K*_total_ *A* also followed a second order polynomial curve, with fitting values of: *B*0 = 0.0546, *B*1 = 2.481, and *B*2 = −0.02098 (*R* ^2^ = 0.9949; Figure 6D). When considering the gas permeability of different storage containers, linear regression analysis showed no significant slope in *K*_total_ *A* for polyolefin (*F* (DFn, DFd) = 2.627 (1, 2), *p* = 0.2465) and PVC‐TOTM (*F* (DFn, DFd) = 2.605 (1, 2), *p* = 0.2479) as agitation frequency increased. However, a significant difference was shown in the intercepts between the two materials (*F* (DFn, DFd) = 3322 (1, 5), *p* <0.0001).

## 4 Discussion

CFD is a powerful tool for characterizing the complex biophysical environment induced by dynamic storage conditions, providing detailed insights otherwise inaccessible through conventional experimental approaches. In this study, CFD was used to simulate PC storage under varying agitation frequencies, to identify how agitation influences mechanical stress and gas–liquid mass transfer. Validation of CFD simulations before interpretation is essential in ensuring the accuracy and reliability of the models’ predictive capabilities. This can be achieved through two primary approaches: (1) by directly capturing the internal fluid motion using particle tracking or imaging velocimetry techniques, or (2) by using the externally observable liquid distribution [22]. Although internal flow measurements provide high‐resolution velocity data, they are often limited by technical complexity, cost, and optical accessibility. In contrast, Method 2 offers an accessible, noninvasive alternative by comparing the predicted fluid motion to the experimentally observable bulk fluid motion. This approach has been widely used in the literature for validating predicted fluid motion in a range of geometries [22, 23, 26, 34, 35].

The CFD model demonstrated strong agreement with the experimentally observed bulk fluid motion induced by oscillatory agitation, successfully capturing nuanced fluid dynamics, such as wave formation, interface deformation, and time‐dependent flow patterns (Figure 2). The model also identified complex behaviors associated with high agitation rates, such as the development and interactions between counter‐propagating waves. These wave interferences, absent at lower agitation frequencies, suggest increased intra‐cycle energy dissipation. Although this could potentially contribute to improved mixing efficiency, facilitating uniform nutrient distribution and improved cell suspension, this could create local regions of elevated mechanical stress or enhanced cell–cell collisions, which could adversely influence PLT activation or viability over prolonged storage.

The influence of agitation frequency on fluid dynamics was further investigated through CFD analysis of velocity fields and WSS at varied agitation frequency (Figures 4, 5). Overall, increasing agitation frequency from 20 to 60 rpm led to marked increases in fluid velocity. At both 40 and 60 rpm, peak velocities were similar and occurred at comparable points in the agitation cycle, demonstrating the preservation of temporal symmetry characteristic of sinusoidal agitation. However, due to differences in cycle times (e.g., 1 s at 60 rpm, 1.5 s at 40 rpm, and 3 s at 20 rpm), the rate of change of velocity was greatest at 60 rpm, suggestive of more dynamic fluid motion per unit time.

The differences in the rate of change of velocity were also reflected by the greater peak WSS, and the spatial distribution of WSS showing more extensive regions of elevated WSS with higher agitation. The averaged WSS predicted in our CFD simulations (1–5 dyne/cm^2^, 0.1–0.5 Pa), while below commonly cited PLT activation thresholds (12–80 dyne/cm^2^, 1.2–8 Pa), are comparable to experimentally measured shear in neonatal PC storage bags [15, 36, 37]. A study by Torres et al. highlighted that increasing the frequency of circular agitation from 3 to 6 rpm increased fluid motion from 1.86 to 3.2 cm/s [27]. With this, a separate earlier study showed that posttransfusion recovery was higher following storage on a 1 rpm circular agitator (43.2%) compared to storage on a 6‐rpm circular agitator (32.4%) [38], suggesting increased WSS through agitation may reduce posttransfusion recovery. Likewise, Pym et al. reported that neonatal PCs experience ∼2.1 dyne/cm^2^ (∼0.21 Pa) at 40 rpm and ∼2.9 dyne/cm^2^ (∼0.29 Pa) at 60 rpm, increasing by ∼0.8 dyne/cm^2^ per 20 rpm increment [15]. Another study showed that introducing “manual mixing” as an alternative to continuous agitation resulted in improved PLT adhesion and reduced GPVI shedding over 5 days of storage [39], highlighting that low‐level, prolonged shear exposure in small‐volume neonatal PCs can contribute to the PSL.

Two container configurations were modeled to investigate O_2_ mass transfer in PC storage: an open top container and an oxygen permeable sealed bag analog. The open top condition was included to (i) provide a validation friendly scenario with free‐surface behavior to compare to experimental results; (ii) to isolate the contribution of unconstrained interface motion; and (iii) establish an upper‐bound case for surface deformation. The CFD simulation demonstrated a nonlinear increase in *K*_total_ *A* with agitation frequency in an open top container (where 1/*K*_total_ = 1/*K*_L_), showing a sharp increase from unagitated storage to 20 rpm, followed by a plateau at higher agitation frequencies (Figure 6). These changes were independent to changes in the gas–liquid interface, suggesting that improvements in mass transfer because of agitation were primarily due to the reduced *T*_c_ between the fluids. According to Higbie's theory, mass transfer occurs during short contact times through the intermittent contact of the interface with fresh fluid elements and is proportional to the square root of the diffusion coefficient by the *t*_C_ (Equation 10). In unagitated storage, the fluid at the gas–liquid interface is largely stationary, resulting in minimal surface renewal and an effectively infinite *t*_C_. As a result, *K*_L_ approaches zero: $\lim_{t_{C}\to \infty }{\left(\frac{1}{t_{C}}\right)}^{0.5}=0$. This means, in unagitated conditions, *K*_L_ is likely governed purely by molecular diffusion. In agitated storage, fluid motion promotes more frequent renewal of interfacial fluid elements, reducing the *t*_C_ between fluids and drastically enhancing overall gas–liquid mass transfer compared to unagitated storage. However, as *K*_L_ is the square root function of *t*_C_, further decreases in *t*_C_ yield smaller changes in *K*_L_, likely reducing the advantage of agitation beyond a certain threshold.

However, when the above is considered alongside the gas permeability of a storage container (where 1/*K*_total_ = 1/*K*_bag_ + 1/*K*_L_), it becomes evident that mass transfer is ultimately limited by the inherent permeability of the storage material. This is demonstrated by both the *K*_total_ and *K*_total_ *A* having no significant dependence on agitation frequency in sealed systems, but instead differ significantly depending on the storage container (polyolefin or PVC‐TOTM). This finding is consistent with the observations made by Pym et al., showing no significant agitation‐dependent differences in internal [O_2_] and oxygen consumption rate (OCR) in stored PCs. Similarly, [O_2_] remained constant over the storage duration, suggesting that once a quasi‐steady state balance between influx and consumption is established, this limitation persists throughout storage [15, 40]. As further increases in agitation show no improvement to *K*_total_ *A*, it raises the question whether *K*_total_ *A* could be more effectively enhanced through mechanisms independent to fluid motion. Adjustments to the storage container geometry or permeability might provide an alternative method to improve O_2_ availability without inducing biophysical cues detrimental to PC quality.

The CFD simulation used in this study modeled the fluid motion of a fixed VOF confined in a rigid body container subjected to flatbed agitation. Although this approach allows for controlled analysis of agitation‐induced fluid dynamics, it may not completely replicate material characteristics of the storage container. Storage containers are made from flexible materials, such as plasticized polyvinyl chloride or polyolefin [41]. This flexibility may introduce fluid–structure interactions, such as wall deformation in response to fluid motion. However, it is important to note the amplitude and frequency of agitation used in PC storage is relatively low, meaning container deformation would be minimal and unlikely to significantly alter bulk fluid behavior and WSS predictions. Additionally, the simulation domain primarily accounts for fluid interaction with the base and lateral walls of the container. In PC storage, the fluid can also interact with the upper surface of the bag, which could introduce cellular rotation absent in the free surface model or influence WSS distribution. Likewise, the top surface of the bag is free to vibrate, which could influence fluid motion and thereby WSS. Although the inclusion of the top wall might refine WSS predictions, it is unlikely to materially affect the identification of dominant flow patterns, or the broader interpretation of mechanical forces generated solely by agitation, which are the primary focus of this study.

Another consideration for the presented simulations is the influence of potential thermal gradients. Although the assumption of a uniform storage temperature (22°C) is representative of controlled storage environments, temperature nonuniformities might arise during PC transit [42]. Although modeling the product container during transport was beyond the scope of this investigation, it is important to recognize that temperature deviations can initiate buoyancy‐driven flow, specifically Rayleigh–Bénard convection, which generates vertical circulatory cells and potentially enhances mixing [43]. The impact of these gradients is especially sensitive in small enclosures, where even modest thermal changes are sufficient to modify internal flow structures, local shear rates, and mass‐transfer dynamics [44, 45]. Transport‐related natural convection therefore might represent an important consideration for future work.

It is also important to note that PC exists as a cellular suspension rather than a homogeneous fluid. In principle, suspended cells could alter bulk rheology or modify flow structures through particle–fluid interactions. However, under the present agitation conditions, the Stokes number ($\mathrm{St}=\frac{\rho_{\mathrm{PLT}\cdot }d_{\mathrm{PLT}}^{2}}{18\mu \cdot T}$), a dimensionless number that describes how particles act under fluid motion, is expected to be ≪1 [46], where *ρ*_PLT_ represents PLT density, *d*_PLT_ represents the PLTs diameter, *μ* is the dynamic viscosity of the fluid, and *T* is the characteristic timescale of the oscillatory flow. This low Stokes number indicates that the PLTs are likely to follow the carrier fluid motion without significantly perturbing it; therefore, the PLTs presence is unlikely to significantly influence bulk fluid motion. Although future models that incorporate predicted particle motion could provide more accurate descriptions of local shear acting on individual cells.

Additionally, the present study assumed laminar flow for all CFD simulations. Although localized transitional or turbulent regimes may arise at higher agitation frequencies, particularly near walls or sharp velocity gradients, this modeling choice was supported by the Re being <2300, based on estimated fluid velocities (≤0.45 m/s), characteristic container dimensions, and fluid properties (density: 997 kg/m^3^; viscosity: 1cP). Likewise, prior research in oscillatory multiphase CFD systems has shown that applying turbulence models, such as Reynolds averaged Navier–Stokes (RANS) based *k*‐epsilon (*k*–*ε*) or *k*‐omega (*k*–*ω*), can introduce numerical dissipation that smooths velocity gradients and dampens interfacial motion, leading to underestimation of peak shear and surface dynamics. This is due to the turbulence kinetic energy (*K*) in RANS equations being damped quickly by the turbulence dissipation rate (*ε*). Furthermore, Mahfoze et al. demonstrated that laminar VOF simulations of sloshing flows more accurately captured interface behavior compared to turbulence modeled cases, which systematically underpredicted free‐surface oscillation amplitudes due to artificial viscosity [47]. Likewise, Mehmood et al. used a laminar VOF model to successfully simulate power dissipation and volumetric mass transfer coefficients in shaken flasks of *Streptomyces pristinaespiralis*, capturing key fluid mechanical drivers of antibiotic production [24]. Taken together, these studies support the use of a laminar model as a conservative yet informative approach to characterize the upper‐bound mechanical stress environment in PC storage. Although it is acknowledged that this approach may underpredict localized turbulence‐driven stresses, particularly at high agitation frequencies, and future studies might consider using hybrid solvers that combine RANS and large‐eddy simulation (LES) approaches to more accurately capture localized turbulence.

Finally, the O_2_ mass transfer modeling in this study was based on Higbie's penetration theory to estimate *K*_L_ based on interface renewal by displacement of the liquid at the gas–liquid interface [48]. This approach is widely used in bioprocessing and bioreactor design due to its strong physical basis and ability to capture dominant features of interfacial renewal without prohibitive computational cost [49]. Although more detailed CFD species transport coupling could provide additional mechanistic insight to the contribution of agitation to O_2_ mass transfer in agitated PC storage, the current model is consistent with other computational and experimental studies reporting limited influence of agitation or shake frequency on O_2_ concentration in mammalian cell systems [15, 23]. This agreement suggests that the present approximation captures the essential features of O_2_ availability under the storage conditions relevant to PC storage.

## 5 Conclusion

To conclude, this study provides a framework for understanding and adjusting the biophysical environment induced by agitation during PC storage. By characterizing the influence of agitation on fluid behavior, this work highlights the potential for CFD to inform evidence‐based adjustments to storage protocols aimed at reducing mechanical stress in PC storage while maintaining O_2_ availability. Although this study modeled geometries representative of small‐volume PCs for neonatal use, recent evidence indicates that such units are valid surrogates for standard adult components when assessing storage‐induced changes [50], supporting the extension of these insights beyond neonatal applications. The findings demonstrate that agitation significantly influences fluid motion, increasing fluid velocity and WSS, while preserving temporal symmetry inherent to sinusoidal motion. Given that mass transfer is limited by the permeability of the storage material opposed to fluid motion, reducing agitation may serve as an effective strategy to minimize the shear induced PLT storage lesion without compromising O_2_ availability.

More broadly, these simulations highlight opportunities for translational optimization of PC storage systems. CFD‐derived parameters could guide new bag‐design criteria, such as optimization of container geometry to limit high‐shear regions, or polymer‐selection strategies that enhance O_2_ permeability while maintaining mechanical robustness. Integrating CFD analyses into regulatory design‐validation pipelines (e.g., as a preclinical tool alongside manufacturer testing and in vitro PC quality assays) would enable manufacturers and blood services to evaluate the fluid‐mechanical consequences of design modifications before clinical deployment. Future work should therefore prioritize combining CFD‐informed design with empirical assessments of PC quality to develop storage protocols and container architectures that better preserve PC function and improve transfusion outcomes.

## Author Contributions

D.P. designed the research study, performed the research, acquired and analyzed the data, and wrote the first draft of the manuscript. A.D., J.O., C.S., C.G., and P.J. supervised the research and reviewed and edited the manuscript. A.M.J. reviewed and edited the manuscript.

## Funding

Dean Pym received a scholarship jointly funded by the Welsh Blood Service and Cardiff Metropolitan University. The other authors received no other specific funding for this work.

## Conflicts of Interest

The authors declare no conflicts of interest.

## Supporting information




**Supporting File**: biot70177‐sup‐0001‐SuppMat.docx.

## Data Availability

The authors affirm that all data supporting the findings of this study are included in the article.
