# Supplementary material for: Computational Fluid Dynamic Analysis of Fluid Motion and Volumetric Gas–Liquid Mass Transfer in Agitated Platelet Concentrate Storage
Source: Biotechnol J. 2026 Jan 8;21(1):e70177. doi: 10.1002/biot.70177 (PMC12848328; doi:10.1002/biot.70177)
Supplement: Supplementary file 1 — Supporting File: biot70177‐sup‐0001‐SuppMat.docx. [file BIOT-21-e70177-s001.docx]

**Supplementary materials**

**S1. Mesh independence study**

The coarsest grid (Mesh I) consisted of 2,070 nodes and 1,540 elements, while the finest (Mesh IIII) contained 477,200 nodes and 452,400 elements. Intermediate resolutions included Mesh II (11,421 nodes, 9,568 elements) and Mesh III (72,345 nodes, 65,520 elements; **Figure S1A**). Mesh quality metrics confirmed that all grids satisfied established standards: the minimum orthogonal quality was 1.0 for all cases, and maximum aspect ratios remained below 5, indicating that mesh distortion did not compromise solution accuracy (**Figure S1B**). Mesh refinements were also shown to improve the resolution of the liquid-air interface. Coarse grids (Mesh I–II) exhibited visible smearing due to numerical diffusion, resulting in poorly defined phase boundaries. Mesh III provided a sharper transition, while Mesh IIII yielded the most accurate interface definition (**Figure S2**).

To assess the impact of mesh density on hydrodynamic predictions, simulations were performed at 60 rpm using Mesh I-IIII. Parameters evaluated included: (**A**) volume-averaged velocity, (**B**) interfacial area-averaged velocity, (**C**) volume-averaged wall shear stress (WSS), and (**D**) interfacial area. Convergence was observed by Mesh III, with negligible differences between Mesh III and Mesh IIII, indicating that further refinement did not affect the results (**Figure S3**). At 40 rpm agitation, similar trends were observed. Mesh I-II produced greater fluctuations in fluid parameters, whereas Mesh III and Mesh IIII exhibited nearly overlapping profiles, confirming convergence at this mesh resolution. Interfacial dynamics were particularly sensitive to mesh density, but stabilization occurred from Mesh III onwards (**Figure S4**). At 20 rpm, coarse meshes (Mesh I-II) showed evidence of numerical diffusion at the interface, while Mesh III and Mesh IIII provided consistent results with negligible differences (**Figure S5**).

**Figure S1. Meshing strategy for meshing independence study.** (A) Structured meshes used in the grid independence study, ranging from coarse (Mesh I) to very fine (Mesh IIII).
(B) Mesh quality metrics including number of nodes and elements, minimum orthogonal quality, maximum aspect ratio, and qualitative assessment. All meshes satisfied recommended quality thresholds, with progressive refinement from coarse (Mesh I) to very fine (Mesh IIII).

**Figure S2. Influence of mesh density on Interface sharpness.** Volume fraction contours of the liquid-air interface obtained with Mesh I-IIII. Coarser grids (Mesh I–II) exhibited pronounced numerical diffusion, while Mesh III produced a sharper interface. Mesh IIII yielded the sharpest and most stable phase boundary with minimal smearing.

**Figure S3. Influence of mesh resolution on key fluid parameters at 60 rpm.** Fluid parameters including (A) volume averaged velocity (B) area averaged interfacial velocity (C) volume averaged WSS and (D) area averaged interfacial area were obtained over five oscillation cycles using Mesh I (green) Mesh II (blue) Mesh III (red) and Mesh IIII (black).

**Figure S4. Influence of mesh resolution on key fluid parameters at 40 rpm.** Fluid parameters including (A) volume averaged velocity (B) area averaged interfacial velocity (C) volume averaged WSS and (D) area averaged interfacial area were obtained over five oscillation cycles using Mesh I (green) Mesh II (blue) Mesh III (red) and Mesh IIII (black).

**Figure S5. Influence of mesh resolution on key fluid parameters at 20 rpm.** Fluid parameters including (A) volume averaged velocity (B) area averaged interfacial velocity (C) volume averaged WSS and (D) area averaged interfacial area were obtained over five oscillation cycles using Mesh I (green) Mesh II (blue) Mesh III (red) and Mesh IIII (black).

**S2. Residual Analysis**

To evaluate for systematic bias, residuals were plotted against agitation rate (Figure S6A). This revealed a random distribution centred around the zero line, indicating that model accuracy was independent of the tested agitation speeds. Model predictive accuracy was subsequently evaluated by correlating the regression model's predicted values with the CFD data (Figure S6B). The data points exhibited strong alignment with the 1:1 reference line, demonstrating high fidelity between model predictions and observed results. Furthermore, the assumption of normally distributed errors was visually assessed using a Quantile-Quantile (Q-Q) plot (Figure S6C). The close proximity of the residual quantiles to the theoretical straight line provided visual evidence of normality. This was quantitatively confirmed by both a runs test (for randomness) and the Shapiro–Wilk test (for normality), which yielded P>0.05. Collectively, these results confirm the residuals are random and normally distributed, thereby validating the statistical robustness and reliability of the CFD model.

**Figure S6.** **Residual analysis of CFD results**. (A) Residuals plotted against agitation rate (rpm), showing random distribution around zero, confirming no systematic bias across tested speeds. (B) values predicted by regression analysis versus actual values showing strong alignment along the 1:1 line. (C) Q-Q plot - Predicted versus actual residuals indicating symmetry and homoscedasticity.
